# Information-Seeking Behaviours of CALD Women with Endometriosis in Australia: A Qualitative Study

**DOI:** 10.3390/ijerph23010134

**Published:** 2026-01-22

**Authors:** Deniz Senyel, James H. Boyd, Melissa Graham

**Affiliations:** Department of Public Health, School of Psychology and Public Health, La Trobe University, Melbourne 3086, Australia

**Keywords:** health information, patient education, endometriosis, women’s health, CALD, cultural diversity

## Abstract

**Highlights:**

**Public health relevance—How does this work relate to a public health issue?**

- Endometriosis affects approximately one in seven women in Australia.
- The condition is associated with substantial personal, social, and economic burdens.

**Public health significance—Why is this work of significance to public health?**

- Understanding information-seeking behaviours is necessary to tailor health information to diverse needs and preferences, particularly among CALD populations.
- Patients’ information-seeking preferences, such as consulting healthcare practitioners, should be strategically leveraged to strengthen patient education delivered by health professionals.

**Public health implications—What are the key implications or messages for practitioners, policy makers, and/or researchers in public health?**

- To form part of a multi-modal informational support approach, centralised, accessible, and easy-to-navigate online information resources should be developed to better support women with endometriosis.
- Improved endometriosis-specific knowledge and training among healthcare practitioners is needed to improve patient education by healthcare providers.

**Abstract:**

Endometriosis affects one in seven women in Australia and is a significant public health concern. Access to appropriate health information is essential for informed decision-making and quality of life, especially for culturally and linguistically diverse (CALD) women who may face additional communication and health literacy barriers. This study explored the information-seeking behaviours and experiences of CALD women living with endometriosis using semi-structured interviews. Through convenience and snowball sampling via social media, eleven women were recruited. Data were analysed using thematic analysis. The results showed that although women often did not view their cultural background as influential, taboos and stigma can shape information-seeking behaviours. Women primarily relied on healthcare professionals, online resources, and other women with endometriosis as information resources. Healthcare professionals were appreciated for providing tailored information, but some were perceived to have limited knowledge of endometriosis, reducing their usefulness. Online information was abundant and easily accessible but often overwhelming and difficult to navigate. Information from other women with lived experience provided both practical insights and validation, though participants recognised its limited transferability to their own circumstances. These findings highlight the need for information pathways, including better patient education through healthcare providers, as well as accessible and evidence-based online resources.

## 1. Introduction

Australia is a culturally diverse country with 28% of the population born overseas and 23% speaking a language other than English at home [1]. Despite this, culturally and linguistically diverse (CALD) communities face multiple disadvantages regarding their health and wellbeing, making them a priority population in the Australian National Strategic Framework for Chronic Conditions [2]. Multiple barriers have been identified that inhibit CALD communities from accessing healthcare services, including low health literacy, language, and communication barriers [3,4,5,6], and equally systemic barriers, such as navigating a complex and foreign healthcare system and the lack of culturally appropriate services [6]. These barriers can be compounded by gender-specific issues, such as the lack of female-specific services that meet cultural needs [7,8]. Symptoms affecting menstruation, fertility, or sexual health are often associated with stigma, which can be amplified by cultural norms and beliefs [9,10]. Therefore, CALD women might hesitate to seek help or discuss their health issues [11]. Consequently, women with menstrual health-related chronic conditions might not receive the support needed to maintain or improve their wellbeing.

One critical chronic women’s health condition, with an estimated prevalence of one in seven women (This article will use the term women to refer to individuals assigned female at birth. The authors acknowledge trans, intersex, and non-binary people living with endometriosis) of reproductive age [12], is endometriosis. It is not only highly prevalent and poses a significant public health issue both internationally and in Australia but also has devastating consequences for those affected. Endometriosis is defined by the growth of tissue similar to the uterine lining outside of the uterus [13] and causes a plethora of symptoms, including dysmenorrhoea, dyspareunia, chronic pain, and infertility [14]. However, even though endometriosis research has gained significant momentum in recent years, the experiences of CALD women with endometriosis in Australia remain largely unexplored. There is a research gap regarding the prevalence, access to care, and quality of life for CALD individuals with endometriosis in the Australian context. International research shows that, while overall the socio-cultural impacts on women with endometriosis are not well understood [15], it has been shown that Black women are less likely to be diagnosed with endometriosis, whereas Asian women are more likely to be diagnosed [16]. This raises the question of whether the underlying prevalence is different or whether women’s experience is based on their ethnicity and cultural changes.

Such experiences become especially important when considering information needs and seeking behaviour. A chronic disease diagnosis is often an antecedent to seeking information [17], yet, similar to accessing health services, this process can be hindered by barriers related to language and culture. Some evidence suggests that there are underlying differences in how CALD and non-CALD people approach information seeking. A study on resources used and trusted regarding child health information showed that CALD parents, in comparison to non-CALD parents, were more likely to trust social media for information and preferred summarised information with bullet points and pictures [18].

While the health literacy of CALD individuals with endometriosis has not yet been studied, evidence from the broader CALD community suggests a correlation between CALD backgrounds and low health literacy [3,4,5]. Thus, it can be hypothesised that CALD individuals with endometriosis may face challenges in accessing, understanding, and applying health-related information [19]. To make existing information resources more culturally inclusive and to design new ones, it is essential to understand the information-seeking habits of women with endometriosis in Australia. Resources must be accessible not only in terms of language, reading level, and comprehensibility but also in addressing the unique needs of people from CALD backgrounds [20,21,22]. This includes understanding the information needs, the language that is preferred, both in a sense of terminology as well as reading level, and lastly, the visual presentation, which can include imagery designed to be inclusive and representative [17,22,23]. The aim of this qualitative study was to explore the information-seeking behaviours of CALD women with endometriosis in Victoria, Australia.

## 2. Methods

A qualitative descriptive design was chosen to enable an in-depth exploration of CALD women’s perspectives on their information seeking, with the intention of remaining close to participants’ accounts to describe the information seeking of CALD women [24]. Ethical approval was granted by the La Trobe University Human Ethics Committee (HEC24359). The manuscript follows the Standards for Reporting Qualitative Research [25].

### 2.1. Sampling, Sample Size, and Recruitment

Given the challenges of recruiting participants from CALD communities [26], a combination of convenience and snowball sampling was used to maximise recruitment [27]. Research shows that for qualitative inductive analyses, the majority of information is gained from the first six to twelve interviews [28,29]; therefore, we set an initial recruitment target of 15 participants to account for the heterogeneity of the study population; however, the primary focus was on achieving thematic saturation. To assess this, data analysis commenced shortly after the first few interviews, enabling ongoing evaluation of the extent to which new data contributed additional insights [30]. Recruitment ceased once no substantial new themes or perspectives relevant to the research question were identified, indicating that thematic saturation had been reached.

Participants were eligible if they were women aged 18 years or older, residing in Victoria, Australia, with either a confirmed or suspected diagnosis of endometriosis. Women were considered CALD if they or one of their parents was born in a non-English-speaking country and/or if a language other than English was primarily spoken at home. Since there is no universal definition of the concept CALD, we adopted a broad interpretation, also including second-generation migrants [31]. This decision was made on the assumption, consistent with recent research findings, that women born in Australia with culturally diverse backgrounds continue to be influenced by those backgrounds [10]. To participate, women needed to have sufficient English proficiency to engage in an informal interview conversation. Participants received an AUD 50 Coles (Australian supermarket) gift card for participation.

Recruitment occurred through personal networks, social media platforms (e.g., Facebook and LinkedIn), and outreach to organisations and healthcare practitioners who were asked to circulate the study invitation. This included local women’s health services and networks, gynaecologists, Primary Health Networks in Victoria, public libraries, and CALD-specific organisations, such as the Ethnic Communities Council of Victoria and the Victorian Multicultural Commission. Additionally, the Health Consumer Centre shared the study through its newsletter and social media.

### 2.2. Data Collection

Data was collected using semi-structured interviews, a commonly applied approach in qualitative descriptive research, as they are helpful for eliciting people’s perspectives on a phenomenon [32]. Women could choose to complete their interview via Zoom or in person; however, all opted for Zoom. While in-person interviews provide richer social and contextual cues that are valuable in qualitative research, online interviews offer practical advantages for both researchers and participants [33], including saving travel time and costs and allowing participants to join from the comfort of their own homes, which can also enhance privacy and facilitate discussion of sensitive topics [34]. Online interviews do require participants to have the necessary technology and digital literacy [33]. However, in this study, no participant was excluded on these grounds, as participants could choose between in-person and online interviews, and all those who chose the online option had the required resources. Privacy was an important consideration for the online interviews. The interviewer conducted all interviews from a private, closed meeting room to minimise the risk of being overheard; however, the level of privacy within participants’ own environments could not be controlled or verified.

The first author conducted all interviews, which were audio-recorded with participants’ re-confirmed consent. The interviewer is a female third-year PhD candidate with experience in qualitative interviewing. As an immigrant to Australia with mixed-race heritage, she brings contextual insight into some of the barriers and challenges faced by culturally and linguistically diverse (CALD) women. However, her educational background, high health literacy, and proficiency in English differentiate her from some participants. Ongoing reflexive consideration was, therefore, undertaken to examine how these positionalities may have shaped the interview process, participants’ disclosures, and the analytic interpretations.

A semi-structured interview guide (Supplementary File S1) was used, featuring open-ended questions exploring the women’s experiences seeking information. Interviews followed a flexible structure, allowing participants’ experiences and narratives to guide the conversation. Prompts and probes were used to elicit further detail.

Interviews were transcribed using Zoom’s automated transcription tool and subsequently edited by the first author for accuracy. One interview had to be excluded due to a faulty audio recording. This was the second interview conducted, and the data was not included. Transcripts were returned to participants for member-checking to confirm the accuracy of the content. The interview length ranged from 27 min to 89 min. The difference in length can be attributed to time since diagnosis and symptom severity, which both impact experiences with information seeking.

### 2.3. Data Analysis

Thematic analysis was conducted using NVivo 14 software following Braun and Clarke’s six-stage framework [35]. The first step involved familiarisation with the data through transcription and repeated reading of the interviews. In the second step, initial codes were generated through line-by-line coding. The codes were reviewed and organised into preliminary themes by identifying patterns of shared meaning, such as the discussion of different information resources. These themes were then refined through an iterative process to ensure internal coherence and distinction, including comparison of individual quotes with the preliminary themes to ensure accurate representative meaning. Final themes were defined and are presented in Section 3, supported by illustrative participant quotes. Each quote includes the participant’s ID and age.

## 3. Results

For the analysis, 11 interviews were included. Only one woman had suspected endometriosis; the rest were surgically confirmed. Two women were second-generation Australians. Two women identified as having an Indian background, while the remaining participants each came from different cultural backgrounds, collectively representing ten distinct (parental) birth countries. Table 1 shows the women’s age, country of birth, the countries of birth of her parents, and year of diagnosis.

Four themes were derived from the data: cultural and linguistic context to information seeking—does it matter?; online information—abundance or overwhelm; healthcare providers as both trusted and limited information resources; and women with lived experience as a source of information and validation.

The first theme, cultural and linguistic context to information seeking—does it matter?, is central to understanding whether and how women’s cultural and linguistic backgrounds shape their information-seeking behaviours. It provides the context for the subsequent themes, which focus on specific information resources.

The remaining themes (online information—abundance or overwhelm; healthcare providers as both trusted and limited information resources; and women with lived experience as a source of information and validation) describe how women use different types of information sources, as well as their strengths and limitations.

**Figure 1:**
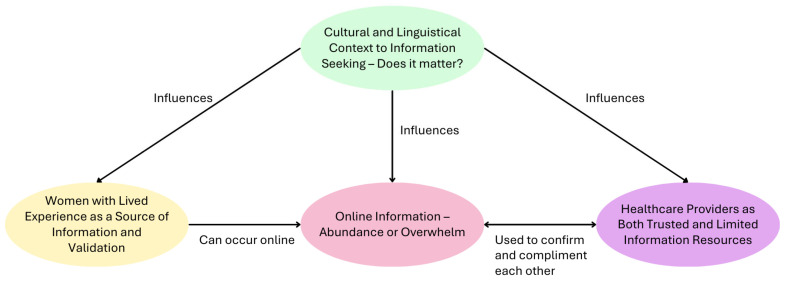
Concept map.

Women did not rely on a single source, and there was considerable overlap between resources. Specifically, the themes online information—abundance or overwhelm and healthcare providers as both trusted and limited information resources are interconnected, as women often used one source to confirm or supplement the other. Figure 1 presents a concept map of the themes, illustrating how they relate to one another. Table 2 offers an overview of the (dis-)advantages of the three information sources: online information, healthcare professionals, and women with lived experience.

### 3.1. Cultural and Linguistic Context to Information Seeking—Does It Matter?

Cultural and linguistic context to information seeking—does it matter? captures women’s perspectives on their information seeking and their cultural background. Women held mixed views on whether their cultural background influenced how they sought information about endometriosis. For some, culture was considered irrelevant; others reflected on how cultural and religious beliefs, menstrual taboos, and stigma could pose as barriers to seeking informational, medical, and social support. These reflections highlight a paradox: while some women rejected the idea that culture shaped their experiences, their accounts nonetheless revealed how cultural and social contexts influenced both their own and their communities’ awareness and openness to discuss endometriosis.

Women expressed mixed views about whether cultural background influenced their search for information. For some, culture was seen as irrelevant; they prioritised information that directly supported them in managing their condition. As one participant explained, “I don’t actually think about my cultural beliefs. I just see what actually works for me” (P05, 23). Others echoed this, emphasising that endometriosis was not connected to culture, and, therefore, some of the women “don’t think endo has got to do [with] anything, or is related to anything with my culture or beliefs or any of it” (P08, 30).

However, one woman, while at first stating that there is no need for culturally adapted information, proceeded to reflect on the ingrained menstrual taboos in India and Nepal and how locally popular media, such as the cinema, can be used to convey information and break down taboos.

“…apart from whether you might translate it into a different language. But other than that I can’t see how you can make it more culturally relevant or culturally really sensitive. […] Although, for example, in India I know there have been, as you might have heard, in India cinema is very popular. A lot of people get a lot of information and entertainment, a lot of things they learn from movies, and there was one a few years ago, which was about a man who was selling in his village, it was based on a true story, sanitary pads. And that was the whole movie was meant to kind of break taboos about menstruation.”

(P07, 31).

This highlights how cultural beliefs may not always be seen as personally influential but are nonetheless recognised as powerful forces in the wider community.

In contrast, some women clearly saw how their culture and religious beliefs influenced them and their surroundings when it came to endometriosis and information seeking. One woman reflected on how cultural and religious beliefs can hinder women from seeking help for their menstrual health.

“That’s also a problem, I think, yeah, not sharing with [their healthcare professional]. Maybe it’s their religious background, the cultural background. I don’t know, language barriers, all those things may be affected.”

(P12, 45)

Taboos around menstruation discouraged women from raising issues with healthcare providers or seeking advice from family and friends. As one put it, “we don’t really talk about this stuff in our culture” (P09, 39). Since some women “don’t want to let people know about the diagnosis” (P03, 27), the consequences can include a lack of support and being left alone with the disease burden. Some women highlighted that the problem extended beyond being taboo to a basic lack of awareness. “In my culture, like my community […] they have zero knowledge about endometriosis” (P08, 30). Therefore, it is hard to say if endometriosis is not talked about because, as a menstrual health topic, it is a taboo, or if there is a baseline lack of awareness on the topic, both of which hinder women from seeking help, a diagnosis, or ongoing support.

Another reason for not wanting to talk about endometriosis was a fear of “stigma” (P03, 27). When asked to elaborate on what P03 meant by stigma, she explained that “a whole lot of people have cultural perceptions with certain things”. This suggests that, in her view, stigma is either caused by perceptions held in specific cultures or at least is correlated with culture and can be heightened in certain communities. Stigma is often rooted in misconceptions and misinformation about conditions. The women voiced a need for better awareness and education to break down taboos, dismantle stigma, and foster open communication. For example, the women believed that “you do have to start with, you know, parents of young women” (P10, 34) and described ways this could be achieved.

“So maybe information [for] parents. If it’s sons, then maybe for dads to talk to them about things like that. And if their daughters, then maybe mum to talk about it.”

(P01, 46).

Other women noted the “lack of health literacy” (P12, 45) in CALD communities and, therefore, the need for “simple language, translations and things like that, a lot of times the community will provide the feedback as well around how they want the message to be done” (P10, 34).

Importantly, the women also cautioned against assuming these challenges were unique to CALD communities. While some identified low health literacy, stigma, and lack of awareness as barriers within their own backgrounds, they also observed similar issues in the wider Australian population. As one woman who had migrated from Sri Lanka reflected, “and even I thought it happened in Sri Lanka, here it’s more people educated about health issues. But here also [has] like [the] same kind of [problem]” (P12, 45).

### 3.2. Online Information—Abundance or Overwhelm

The theme online information—abundance or overwhelm portrays women’s experiences of using online resources for seeking information. These experiences ranged from positive, as online information was seen as abundant, convenient, and accessible, to negative, as online information could also be hard to navigate, authenticate, and not catered to the right literacy level. The (dis-)advantages of online information can be seen in Table 2.

Online resources were seen as an abundance of information, which the women believed had its advantages and disadvantages. The internet contains many different information sources and formats. The women mostly referred to online written information in the form of websites, which range from medical databases and articles, non-government organisations (NGOs), and government websites, as well as general health information pages. Another important aspect was social media platforms. These were used by the women to access information from other women with lived experience and relevant content by healthcare professionals or organisations. The women’s use of social media to seek information from and engage in conversation with other women with lived experience is discussed in the theme women with lived experience as a source of information and validation. This separation is intentional, as the women discussed these interactions in ways that related more to the connection of information seeking and validation, which could occur both online and offline. Social media use beyond lived experience exchanges was discussed less frequently, but where relevant, it has been incorporated into the current theme.

The bandwidth of information described ranged from basic and concise information, such as the one-pagers on endometriosis by Jean Hailes’s (Australian national not-for-profit organisation providing information resources on women’s health), to highly detailed and specific information from medical websites and journal articles. Therefore, online information accessed by the women catered to all health literacy levels and needs. One woman explained why she valued the Jean Hailes website. “Everything’s condensed in one page, and there might be a few simple diagrams or graphs or things that that are meant to explain something. So something that’s yes, very concise.” (P07, 31).

On the other hand, some women did prefer in-depth information. “I mean I’m an academic scientist also to try and find some research articles if possible or just you know articles from say Cleveland Clinic or you know WHO [World Health Organization] or whatever something like that’s reliable.” (P10, 34).

Conversely, if information resources did not meet the women’s literacy level, they could be seen as either of little use or as inaccessible. One woman who works in the healthcare sector and identifies as having high (health) literacy stated that online information is “very basic information for someone who’s got no idea about what’s happening” (P01, 46). However, she also reflected that even if you have the ability to read research papers, it is “very time consuming” (P01, 46) and, therefore, not feasible for all. Another woman explained that she might not use websites if they are hard to understand for her. “What I guess what really matters is the accessibility, how easy it seems for you to be able to access it without stress.” (P05, 23).

While resources for different target groups exist, the sheer amount made it difficult for some to navigate, making the women wonder. “But it is hard sometimes choosing which way do we have to go” (P12, 45). This confusion and lack of guidance on where to find accessible and credible information made women “search anywhere and everywhere” (P11, 29). To counteract this, the women chose websites based on self-described criteria, such as familiarity, “whatever I feel is authentic” (P03, 27), or medical websites, such as “Cleveland Clinic […] WHO” (P10, 34) or “government backed” (P07, 31) websites.

Overall, while most women reported no issues understanding information online, some reported having language difficulties, especially regarding medical terminology. As one woman explained, not understanding a word can lead you into a rabbit hole that strays you from your initial question. “More like uterine fibroid. I checked it some time ago, and they were just giving me types of the fibroids. I was seeing words like myomas leomas words like they keep coming, and you have to click on it to know the definition. So this keeps pulling you from the main information you want. You have to not search for other words.” (P03, 27).

In addition to medical terminology, language can be a barrier for CALD women, even with the availability of translation tools. “Because there are actually diverse kind of languages. And some people might not even understand languages, and would have to make use of Google translation, which can take more time and be stressful, draining and even frustrating.” (P05, 23).

Lastly, online information had some clear advantages, as it was reported to be a cheap, convenient, and accessible alternative to healthcare professionals to receive information. The women explained these advantages by noting that “Google is at home. You can do it anywhere” (P03, 27) and that “it can save costs” (P05, 23). Further, online resources, especially social media, allowed women to access the knowledge of a wider range of healthcare practitioners, information that their own providers did not have time to cover in a standard consultation. As one woman explained, “I’m following a lot more medical practitioners now. And they give some really, really useful tips like so helpful which you don’t get from a practitioner that I see, you know, consultation. Not that they’re not helpful, but it’s just they can’t go through every single thing.” (P09, 39).

These comments illustrate why online information is sometimes preferred, as its benefits directly address the limitations of asking healthcare professionals questions. These limitations are discussed in the theme healthcare providers as both trusted and limited information resources. Additionally, online information had the advantage of being more private and discreet, which was valued for sensitive or taboo topics about which women felt uncomfortable speaking with their healthcare professionals. As one woman explained, online written information is more comfortable than receiving information from a healthcare provider for sensitive topics. “I don’t really mind, but I would prefer reading just to be a little bit more comfortable. Because I will not be able to talk about it completely to a doctor. I can just say a little bit, but obviously not a lot. Yeah. So I would probably prefer reading.” (P01, 46).

While online information offers advantages over asking a healthcare professional, healthcare professionals also have strengths that online resources cannot provide, as discussed in the theme healthcare providers as both trusted and limited information resources. Therefore, online information and information from healthcare professionals were not seen as opposites or separate but rather often used in combination. Using multiple sources was often applied by the women to create a full picture and receive all the information necessary. “Then I can go to all the platforms, and at the end of the day, after combining this and that, I kind of get the information that I actually want.” (P05, 23).

Healthcare professionals were generally considered the most reliable source of information by the women, and as such, some women sought medical advice after reading information online. “I wouldn’t trust the Internet too much […] I would go back to my GP and ask” (P08, 30). Another woman explained how she brought information she found online to her consultation to discuss it with her GP. “Instagram has a lot of dieticians talking about endometriosis. So I follow those pages. I spoke to my GP about it like, I literally printed a list of things I should eat and things I should not eat.” (P08, 30).

### 3.3. Healthcare Providers as Both Trusted and Limited Information Resources

The theme healthcare providers as both trusted and limited information resources describes how the women saw healthcare professionals, particularly general practitioners (GPs), as the main but also an inconsistent source of information about endometriosis. While many of the women valued GPs as trusted first points of contact, others found their knowledge limited, their advice dismissive, or their support inadequate. Table 2 highlights the (dis-)advantages of healthcare professionals as information resources.

For many of the women, their GP was the first healthcare professional they consulted, and the women positioned them as a key information resource. The women felt that when GPs were knowledgeable about endometriosis, they were often the first to raise the possibility of endometriosis and support the women through diagnosis. One woman explained how a GP specialising in women’s health identified her symptoms as possible endometriosis after years of painful periods she had assumed were normal. “She’s the one who’s firstly said, oh, you’ve got endometriosis. I’m like what? I’ve never even thought that I would have it, because that’s pretty much only time. And then she said, Oh, do you have painful menstruation cycle? Yeah, it has been painful for last, say, about 6, 7 years, but I just thought it was just menstruation.” (P07, 31).

By contrast, other women reported years of being dismissed by healthcare providers who failed to offer diagnostic investigations despite women raising concerns about symptoms such as persistent pelvic pain. One woman recalled her GP failing to investigate her symptoms. “I’ve been talking to him about pelvic pain for a long time, and he didn’t really do anything about it” (P01, 46). Another woman recounted her pain being dismissed and having to advocate for herself to receive some diagnostics. “My initial GP, that I had as a teenager, she dismissed me for a long, long time. I had to really push her to get an ultrasound when I was in my teenage years. Because like I said, she just didn’t believe me. And she just kept saying oh just bad period pain.” (P09, 39).

Because of the dismissive attitude of some healthcare providers, the women reported having to “advocate for ourselves and how we can ask for those specific tests or specific supports” (P10, 34).

Information support after diagnosis was also mixed. Some women felt GPs provided only “general advice” (P07, 31) or thought “GPs don’t know a lot” (P09, 39). In many cases, women were simply referred to specialists with little or no further information. For example, one woman reported her GP “was like, oh, I’m going to refer you to a specialist […] They didn’t really give me any leaflet, or any kind of information whatsoever” (P01, 46). Yet, specialist consultations often failed to address women’s broader questions, focusing narrowly on surgical details rather than long-term management. As one woman reflected, “My concern was how do I manage this long term? […] What are the things to look out for?” (P11, 29).

Several women described how time constraints in consultations limited meaningful information exchange. As one woman explained, “GP consultation, like 10 or 15 min, then after that, nothing, like no follow up. Yeah, like no connection […] I felt like they have to educate patient more” (P12, 45). Equally, specialist appointments were seen as expensive and short on time. “I think with the challenges with the specialist is A the cost, but then B, you know you don’t, you feel like you don’t get that much time and then yeah, you get short answers or nonspecific answers and then you come back and then you wonder, you still don’t have your answer. So you know you don’t want to go back again.” (P10, 34).

On the other hand, consultations with healthcare professionals could be highly informative and helpful if enough time was provided. “We spent like over an hour for an appointment. It wasn’t rushed so I could kind of ask all the questions I had questions written down at the time of appointments. And he kind of answered everything.” (P01, 46).

Detailed explanations that are communicated at a pace that women can follow were important, as illustrated by P03. “Bit by bit. I like a detailed explanation. So with me, you have to be very patient, so that I would learn. I would hear you. I would listen to you, and then pick whatever I want to pick, so he was very attentive to details, and he, I mean, whatever he was saying, resonated with me.”

As previously discussed in theme online information—abundance or overwhelm, information resources are rarely used by themselves. Women preferred online resources to supplement consultations, providing content that could be revisited and digested at their own pace. “It takes time to process and if I hear all the information, I’m not gonna have questions straight away, I’m gonna have to go back. Think about it. Process information. Then I’m going to have the questions. So whatever the specialists or the doctors give, I would still prefer it in online I can go back to and read, because it’s impossible to absorb all the information they you can get given.” (P01, 46).

When asked how consultations with healthcare providers could be further improved, women stated that eradicating dismissal and listening to women was vital; “if a patient’s telling you they’re in pain you should believe them” (P09, 39). Instead of dismissal, the women believed that healthcare providers should admit limitations in their own knowledge, which creates trust, as P10 explained. “I feel like I can trust her. I can rely on her. And then, yeah, usually if she doesn’t have an answer and then you know, if she feels like a specialist is needed, she would give the specialist referral.”

Other contributing factors for a good patient–provider relationship included using positive language that gave women encouragement and hope. One woman explained how using positive language mattered to her. “But if you go and you say we might not know what causes endometriosis, but we might have you know, some treatment options that could help you. There’s the things you can do. So the way you deliver your message to you know your participants or your audience is very, very important.” (P08, 30).

Having a positive patient–provider relationship also matters during the information-seeking process. One woman explained how a consultation is not just about conveying information but also about personal connection. “The way we try to spice things up like getting having some jokes in the middle of a conversation, smiling at each other, laughing at little jokes. I think that actually sparks a lot more understanding than just having to seek information about the person you’re trying to get information from.” (P05, 23).

Lastly, positive experiences were often characterised by healthcare providers who took time to explain clearly and tailor information to the woman’s circumstances. “If you go to your specialist or your GP, they’ll be able to make sure that information is tailored to you and that you’re not going to be like overthinking stuff.” (P11, 29).

Tailored information was also important due to the chronic and evolving nature of endometriosis, which makes each woman’s journey with endometriosis unique. GPs who guided women step by step through treatment decisions were described as supportive partners on the “journey” (P11, 29). For example, one woman appreciated her GP’s staged approach to managing her condition before considering hysterectomy, explaining “It kind of went through like stages […] we had about 3, 4 sessions […] when I did have some concerns, I called him. And he was like, Yeah, that’s fine, you know. I’ll tell you answers.” (P01, 46).

Personal factors such as gender and language shaped comfort in healthcare interactions. Many women preferred female practitioners. However, this could be more of a personal choice rather than a cultural necessity, and one woman explained her preferences. “[The choice is] not more about my religion, because we can go [to a male practitioner], it’s not forbidden, we can go, but for me it was more about me” (P08, 30) though they were willing to see male doctors if necessary. Another woman explained that while she does consult male practitioners, she does prefer female practitioners for women’s health specific examinations: “I do have a female practitioner that I would go for pap smear and things like that, because my usual practitioner is a male and I didn’t really feel comfortable.” (P01, 46).

Several women also suggested that a practitioner who spoke their native language would be preferable to an interpreter, both for privacy and ease of communication. “So the practitioner speaks my language that would be a lot more easier. One less person in the room. That means one less person to be embarrassed in front of so definitely, I would prefer practitioner to speak the language. But if not, then interpreter would be of same sex.” (P01, 46).

Finally, the women generally trusted medical professionals and saw no health topics as taboo in consultations, recognising them as spaces where sensitive issues could be openly discussed. This trust extended to friends or relatives with medical training, even if they were not specialists in endometriosis, as their professional knowledge made them reliable sources of advice. One woman recounted being newly diagnosed and calling a friend with medical expertise to discuss the diagnosis. “So you know, I called one of my doctor friends that stays in New Zealand and you know I had to communicate with him. […] He also told me due to the abnormal growths that bruise in the body.” (P06, 35).

However, one woman reflected on her own experiences as a medical practitioner and how she notices that “People just don’t talk about it, especially having sex, pain with the sex” (P01, 46), indicating that some taboos might even exist in a medical setting.

### 3.4. Women with Lived Experience as a Source of Information and Validation

The theme women with lived experience as a source of information and validation captures women’s experiences seeking discourse with other women with endometriosis, both online and in person, to receive information as well as emotional validation. Table 2 illustrates the (dis-)advantages of women with lived experience as information sources.

Women with endometriosis were seen as an important information resource to both understand the condition and its impact it has on women, as well as learning about self-help strategies. One woman, who was newly diagnosed, recounted meeting another woman with endometriosis and found this helpful to understand how the disease might progress and what the future could hold for her. “I think that’s a person I can actually get proper information from, because I’m gonna get to know how she feels every day, even if my own is just starting at the point. And she had already been having the symptoms in her. At least, I need to know what is to become of me, like what and what I’m to face.” (P04, 30).

Speaking to women with endometriosis was not just helpful to understand the condition better but also how to live with it. When asked what the most useful information to her was, a woman answered that practical tips that have worked for others that she could try for herself were the most helpful. “I can see that certain things actually I saw online or certain experience of other people I saw online that I actually tried I incorporated it into my life and some actually worked for me. […] Those are actually the things I didn’t actually get from my doctors. But I actually got online and I’ve been trying it. It has been working for me. So I kind of feel positive about it.” (P05, 23).

As this quote also illustrates, lived experience was an important addition to information from health professionals, as it provided tangible and practical advice on how to manage endometriosis.

At the same time, participants recognised the limits of peer knowledge. Women valued hearing others’ stories, but many were cautious about transferring advice to their own situation, especially when experiences differed in severity or complexity. As one woman explained, her friend’s advice was useful, but “her health situation overall is very different from mine” (P07, 31). Many women stressed that while they sought peer perspectives, they would confirm advice with a GP, seeing medical input as “more reliable” (P08, 30).

For many, listening to lived experience was a way of comparing their own “condition with others’ condition” (P05, 23) and feeling like they are not alone, recognising “like most of them are on the same boat” (P08, 30). The women described receiving daily inspiration and learning how others were “navigating or advocating for themselves” (P10, 34). Talking openly with peers also helped break down taboos, especially around painful sex, and encouraged women to raise these issues with their doctors. “Knowing that other people are also feeling that […] it’s more like a reassurance that I’m not alone in the world. And it’s okay to talk about it to the doctors as well.” (P01, 46).

Therefore, even when the intention was just to seek validation and compare their situation to others, women gained information on symptoms and what to discuss with healthcare providers.

Women connected with other women with endometriosis both online and in-person. In-person contact was recounted less but could be very impactful, as one woman recalled that meeting someone “still surviving” gave her the strength to keep on living. She described the mental battle she fought, before meeting someone else with endometriosis, as follows: “Those are negative like thoughts I have in me that I’m gonna pass away […] I just have to wave it off and say, no, I’m going nowhere. I’m gonna fight this, I’m gonna survive it.” (P04, 30).

Since not many women knew others with endometriosis in their lives, online platforms, such as Instagram and Facebook, were important spaces for connection. Some engaged passively by reading social media posts by women with lived experience “to learn new things” (P05, 23). This could be highly impactful on women’s mental health, as one woman explained. “I try to follow some people on my Instagram with endometriosis. You know some people that are survivors. Most of them did surgery. Most of them still live with it you know. I just follow them like they give me inspiration every day, a reason to be alive.” (P06, 35). Other women actively shared their stories, finding empowerment in “talking about it from my perspective […] as an endometriosis warrior. (P09, 39).

These online spaces were especially valuable for women who lacked support offline, whether because their family dismissed their symptoms or friends had little knowledge of endometriosis. As one woman described, the absence of understanding from her social circle compounded the mental health burden. “I was going through a really hard time processing everything and I didn’t have a lot of friends that I could talk to about my health […] a lot of people didn’t understand me.” (P09, 39).

Social media was one way to find others with endometriosis and to fill that gap. To summarise, connecting with women with lived experience, either in-person or online, was an important resource not just for information on the condition and how to live with it but also for camaraderie, hope, and inspiration.

## 4. Discussion

The aim of this study was to explore the information-seeking behaviours of CALD women with endometriosis in Victoria, Australia. The findings highlight the divergence of women’s experience in relation to whether or how their cultural background influenced how they sought information. While many initially stated that culture did not matter, they also described how taboos, stigma, and a general lack of awareness about endometriosis were embedded in their communities. As such, cultural norms could act as barriers to seeking both professional and informal advice. For their information seeking, the women mainly used three particular information sources: healthcare professionals, online resources, and other women with lived experience of endometriosis. Healthcare professionals were generally trusted and valued for providing tailored information; however, it was perceived by the women that some lacked sufficient knowledge about endometriosis, limiting their usefulness as information resources. In contrast, online information was abundant and easily accessible, but its sheer volume could be overwhelming and make it difficult to identify reliable sources. Online information and healthcare professionals were not consulted independently; women described using a combination of these resources, cross-referencing information to verify accuracy and fill gaps. This strategy allowed them to build a more complete understanding of their condition in the absence of consistent or comprehensive guidance from any single source. Other women with lived experience offered both information and validation, though women recognised the limits of applying others’ experiences to their own situations. Overall, women navigated a complex information landscape by drawing on multiple sources simultaneously, using each to compensate for the limitations of the others, and fulfilling their information needs. Their cultural and linguistic background may not always be consciously acknowledged by women themselves, yet internalised taboos, stigma, limited community awareness of endometriosis, and language barriers nonetheless influenced their information-seeking behaviour.

While healthcare providers have not been described as information sources in the literature, a lack of knowledge among healthcare professionals on endometriosis, especially GPs, has been highlighted [36,37,38,39]. This underlines the need for better education for healthcare providers, as, according to our study results, the women would like to receive information on endometriosis from their healthcare providers. Currently, in Australia, information and education are available to healthcare professionals via the Royal Australian and New Zealand College of Obstetricians and Gynaecologists (RANZCOG) and the Royal Australian College of General Practitioners (RACGP). Additionally, both national and international guidelines exist on the diagnosis and treatment of endometriosis [40]. These resources need to be promoted more among healthcare providers to enable greater utilisation and to improve healthcare providers’ knowledge of endometriosis. Promotion could occur through established professional education pathways, including continuing professional development programs, clinical newsletters, and targeted dissemination through Primary Health Networks.

Online communities emerged as an important source of validation and informal advice in our findings. Research on the information-seeking behaviour of CALD women has not been previously conducted. However, comparisons with the general endometriosis community can be drawn. An Australian study found that 76% of women with endometriosis used social media to seek information [41]. Research exploring various platforms, such as Reddit [42,43] and Facebook [44], supports these findings, showing that women use social media and online support groups [45] not only to exchange experiences but also to access different types of support [44]. This includes informational support (advice or guidance), emotional support (empathy and reassurance), esteem support (affirming strengths and confidence), network support (fostering social connection), and tangible support (practical assistance) [46]. However, much of the existing research focuses on analysing social media content rather than exploring women’s own perspectives on using these platforms [42,43,47,48,49]. Similarly, while numerous studies have examined the quality of online health information [50,51,52], fewer have investigated women’s information-seeking behaviours directly. One such study conducted by Sbaffi and King [53] with an international cohort identified patterns consistent with our findings, with women cross-referencing multiple sources and favouring trusted sites, such as national endometriosis organisations and medical institutions. In contrast, an Italian study by Arena et al. found that younger women solely used the internet for receiving information on endometriosis [54]. Overall, our findings on the information-seeking behaviour of CALD women with endometriosis overlap with the limited current evidence available.

Stigmatisation and taboos surrounding endometriosis have long been identified as barriers to help-seeking and receiving care for women [9,55]. Beginning in classrooms, where menstruation is treated as taboo and period pain is normalised, this extends into adulthood, where women may feel inadequate at the workplace, as their leave of absences caused by endometriosis might be frowned upon [56,57]. As a result, symptoms such as pain may be ignored due to internalised normalisation, and women may feel ashamed to talk about symptoms such as pain during sex because of the associated taboo [56]. As international evidence shows, stigmatisation of endometriosis and the taboo surrounding the topic affect women from diverse backgrounds, not only those from specific cultural communities [9,55,56]. However, cultural norms and perspectives on menstruation can contribute to and further compound this stigmatisation and taboo [58,59]. While it is well established that these factors affect women’s access to healthcare, including diagnosis and care, the ways in which taboos and stigmatisation influence information-seeking among women with endometriosis have not yet been adequately explored.

As the first study to examine information-seeking among CALD women with endometriosis, our findings introduce an important cultural dimension to understanding how women navigate information. Cultural sensitivities, stigma, and taboos shaped women’s preferences, with some favouring female practitioners or the anonymity of online spaces for discussing certain topics. While most studies focus on a single information source, such as the internet, they overlook the interplay between different resources. Our findings highlight the interconnected nature of information seeking, particularly the way women combine online sources with guidance from healthcare professionals. Moreover, while previous research often examines women with lived experience primarily as providers of emotional support, their role as sources of informational support has received less attention.

### 4.1. Limitations and Strengths

This study has several limitations. Women were eligible only if they spoke sufficient English, which excluded those who might face the greatest challenges due to language barriers. This may explain why women in this study generally felt comfortable accessing and researching information in English. The inclusion criterion was chosen to protect participants’ privacy, as the presence of an interpreter could make the interview setting less personal. Women themselves described feeling uncomfortable when interpreters were present during healthcare consultations. Additionally, translating interview transcripts could have introduced misinterpretations or loss of nuance. We, therefore, acknowledge that women with limited English proficiency may have been excluded from this study, which could have biased the findings, as their experiences may have differed from those of our cohort.

Secondly, women who viewed endometriosis as a taboo topic may have been less likely to volunteer for participation. The women in this study appeared comfortable discussing their experiences and did not express strong internalised stigma, shame, or discomfort. However, they provided valuable insights into how taboo and stigma operate within their broader communities. While this study could not capture the full diversity of cultural and linguistic backgrounds or experiences of all CALD women with endometriosis, it represents one of the first attempts to explore their information-seeking experiences and highlights the variation in information needs across this heterogeneous population.

Finally, the online setting may have constrained rapport building and, in turn, the depth of discussion. However, because participants were able to choose their preferred interview setting, it is likely that the online format enhanced their comfort and engagement.

This study also offers several strengths. First, it is one of the first studies to examine the information-seeking experiences of CALD women with endometriosis, providing novel insights into a population that is often underrepresented in endometriosis research. By considering cultural and linguistic context, the study advances understanding of how these factors shape the ways women seek information. Secondly, the use of semi-structured qualitative interviews allowed for rich, detailed accounts of women’s information pathways, capturing the interplay between online resources, rather than limiting discussion to a single source. Finally, by including participants from diverse CALD backgrounds and adopting a broad definition of the term, the study identifies overarching patterns that can inform practical improvements, such as allowing more time during consultations to ensure comprehension and providing online resources in multiple languages.

### 4.2. Recommendations and Future Work

The findings demonstrate that no single information source is sufficient to meet women’s diverse information needs. Information seeking occurs across multiple platforms and literacy levels, emphasising the importance of offering information through multiple channels. Healthcare professionals remain highly trusted and valued information sources on endometriosis. To strengthen their role, consultation times, especially following the endometriosis diagnosis, should be extended to allow for questions and discussion, particularly for CALD women who may face language barriers. Having enough time during consultations and a good patient–provider relationship were identified as essential components of a safe and supportive environment. To meet these expectations, healthcare providers, especially GPs, may require improved education about endometriosis and how to better provide patient education. Since the women reported that their GPs did not provide information and were only referred to specialists whom the women believed also lacked guidance, there is a need to clarify responsibility for delivering patient information and to improve communication between GPs and specialists. Where appropriate, healthcare providers should direct women to trustworthy, accessible, and literacy-appropriate information sources. Some positive examples from the interviews included the Jean Hailes website in Australia or the Cleveland Clinic website from an international perspective. Both offer information pages on endometriosis.

For online information, providing translations and simplified English versions is critical to address varying levels of health literacy. Additionally, “Easy Read” versions can make information accessible for women who face language barriers or who have low literacy. For example, our results showed that combining text with imagery can make information resources easier to understand and more appealing. Guidelines on how to design “Easy Read” resources provide standards to support the development of material that meets women’s needs [60]. Even women with high literacy often lack the time or background knowledge to interpret complex medical literature, underscoring the need to translate research findings into plain language suitable for a lay audience.

Future research should aim to address language barriers and engage CALD women experiencing stigma and taboos around menstrual health. Collaborations with bilingual healthcare professionals and community workers may help reach these women more effectively. Further studies are needed to better understand CALD women’s journeys with endometriosis from diagnosis to treatment and how cultural and social contexts shape their access to and use of information.

## 5. Conclusions

This study highlights that CALD women with endometriosis draw on a diverse range of information sources to understand and manage their condition. While healthcare professionals remain a valued and trusted source, inconsistent knowledge and dismissive attitudes can undermine their credibility and drive women towards alternative resources. Therefore, a better healthcare provider is needed in order to provide better patient education. Online platforms and lived experiences, therefore, play an essential role, not only in filling informational gaps but also in providing emotional validation and a sense of community. However, the abundance of information available online can be overwhelming and requires women to critically evaluate and cross-reference sources. Patient education by healthcare providers can help guide women to evidence-based and relevant resources. Cultural and social influences, including stigma and taboos, further shape how and where women seek information, often constraining open discussion and help-seeking within their communities. Overall, these findings underscore the need for accessible, culturally sensitive, and evidence-based information pathways that empower women to make informed decisions about their health and navigate endometriosis with confidence.

## Figures and Tables

**Table 1:** Participant demographics.

ID	Age	Participants’ Country of Birth	Participants’ Parents’ Country of Birth		Languages Other than English Spoken at Home	Diagnosis Year
			Mother	Father		
P01	46	India	India	India	Gujarati	2023
P03	27	Somalia	Somalia	Australia	Somali	2023
P04	30	Ghana	Ghana	Ghana	Twi	2019
P05	23	South Africa	Australia	South Africa	-	2020
P06	35	Togo	Togo	Togo	Native tongue (not further specified)	2017
P07	31	Australia	India	India	Punjabi	-
P08	30	Jordan	Jordan	Iraq	Arabic	2024
P09	39	UK	Malaysia	Malaysia	Tamil	2017
P10	34	Nepal	Nepal	Nepal	Nepali	2024
P11	29	Australia	Australia	Lebanon	Arabic	2024
P12	45	Sri Lanka	Sri Lanka	Sri Lanka	Sinhala	2008

**Table 2:** (Dis-)advantages of information sources.

Information Resource	Advantages	Disadvantages
Online information	Convenient, accessible, low-cost, and available anytime and anywhere Provides a wide range of information, from basic overviews to detailed scientific content Allows women to seek information privately, particularly for sensitive or taboo topics Enables women to revisit information and process it at their own pace	Overabundance of information can be overwhelming and difficult to navigate Difficult to assess the credibility and authenticity of sources Information may not match women’s health literacy levels Medical terminology and a lack of translation can create a language barrier
Healthcare providers	Generally perceived as a reliable and trustworthy source of information Trust in medical expertise Can provide personalised information Opportunity to ask questions	Lack of knowledge on endometriosis Experiences of dismissal and disbelief undermine trust Cost and short appointment times limit usefulness and accessibility
Women with lived experience	Provide practical, experiential knowledge about living with endometriosis Offer emotional validation Help normalise symptoms and break taboos	Experiences are highly individual and not always transferable

## Data Availability

The original contributions presented in this study are included in the article. Further inquiries can be directed to the corresponding author.

## Supplementary Materials

The following supporting information can be downloaded at https://www.mdpi.com/article/10.3390/ijerph23010134/s1, Supplementary File S1: Interview guide.
