# Supplementary material for: Information-Seeking Behaviours of CALD Women with Endometriosis in Australia: A Qualitative Study
Source: Int J Environ Res Public Health. 2026 Jan 22;23(1):134. doi: 10.3390/ijerph23010134 (PMC12840965; doi:10.3390/ijerph23010134)
Supplement: Supplementary file 1 [file ijerph-23-00134-s001.zip › ijerph-4081338-supplementary.pdf]

Interview

1. Can you tell me about your current needs for information relating to endometriosis?

2. Can you tell me about your experience of looking for and accessing information on endometriosis?

3. Imagine you would design your ideal information resource, what would it look like?

4. Is there anything else you'd like to share today about your experiences or information needs?

Exemplary probes:

- Can you tell me more about that?
- Why do you think that is?
- How did that make you feel?
- Can you give me an example?
- What do you mean by that?
- Could you clarify that for me?
- How did you handle that situation?
- What were the challenges you faced?
- How did you come to that decision?
- What was going through your mind at that time?
- What were the key factors in that?
- In what way was that important?
- How would you describe that in more detail?
- What other options did you consider?
